# Supplementary material for: Bridging Gaps in HDR Improvement: The Role of MAD2L2, SCAI, and SCR7
Source: Int J Mol Sci. 2023 Apr 4;24(7):6704. doi: 10.3390/ijms24076704 (PMC10095018; doi:10.3390/ijms24076704)
Supplement: Supplementary file 1 [file ijms-24-06704-s001.zip › ijms-2333772-supplementary.pdf]

Supplementary Materials

Selection of siRNA for MADL2 knockdown

Three small interfering RNAs (siRNAs) were designed for the NM_001127325.2 isoform of *MAD2L2* that consists of 8 coding exons and encodes a 211 aa protein. The siRNA_MAD2L2_1 sequence is located at the border of the first (non-coding) and second exons of the transcript, siRNA_MAD2L2_2 and siRNA_MAD2L2_3 are both complementary to the last exon (Figure S1). These two siRNAs have deoxythymidine residues at the 3'-end of the strand, and siRNA_MAD2L2_1 has a double uracil residue. Transfection of siRNA was performed with the TurboFect™ Transfection Reagent (Thermo Fisher Scientific, USA). Cells were analyzed 24 h after transfection.

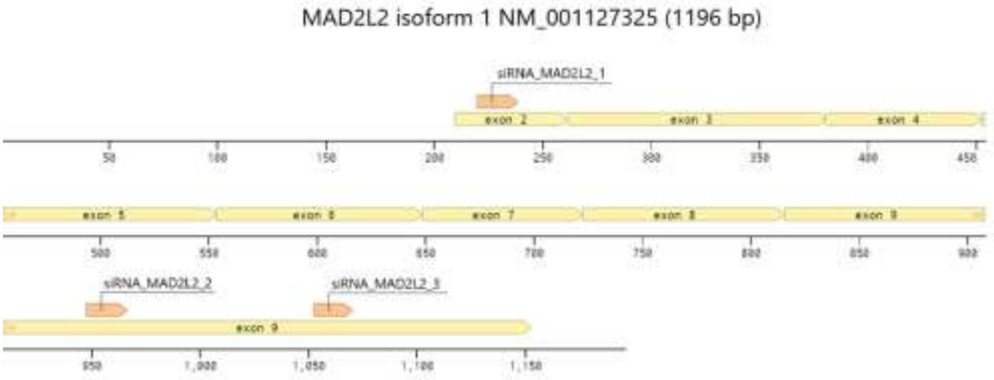

Figure S1. MAD2L2 and anti-*MAD2L2* siRNAs. First exon is non-coding and isn't shown.

Transfection efficacy (97.1%) was measured using a FAM-labeled siRNA (siRNA_flu) with a sequence that was non-specific for the human genome. Negative controls included untransfected cells, cells with transfection reagent only (Turbofect), and cells transfected with siRNA_flu. MAD2L2 expression in the cells transfected with siRNA_flu was set to 1 and othe samples were compared to it. Cells at all conditions showed the same normal survival 24h after transfection (data not shown). All 3 anti-MAD2L2 siRNAs demonstrated expression knockdown down to 0.3 ($p=0.012$), 0.09 ($p=0.017$), and 0.1 ($p=0.017$) compared to MAD2L2 expression in the cells transfected with siRNA_flu (Figure S2). siRNA_MAD2L2_2 demonstrated more than 10-fold knockdown and was selected for further experiments; it is called as siRNA_MAD2L2 in the main text.

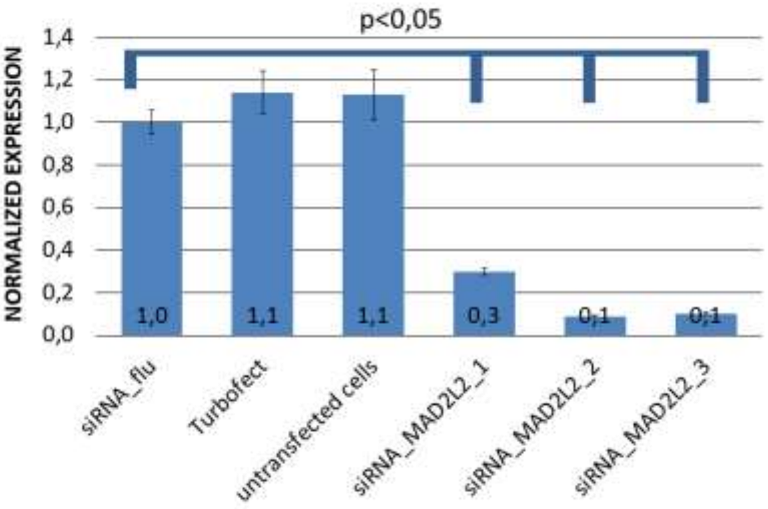

Figure S2. Expression of *MAD2L2* mRNA. Data is represented as mean and 95% CI, differences in expression levels were analyzed using the Mann-Whitney test. More details are in the text.

We also confirmed that *MAD2L2* influences its protein level. We stained the cells with anti-MAD2L2 antibodies and demonstrated that the number of MAD2L2 positive cells decreased 1.8-fold 24 h after transfection compared to control samples exposed to Turbofect only (S3 Fig).

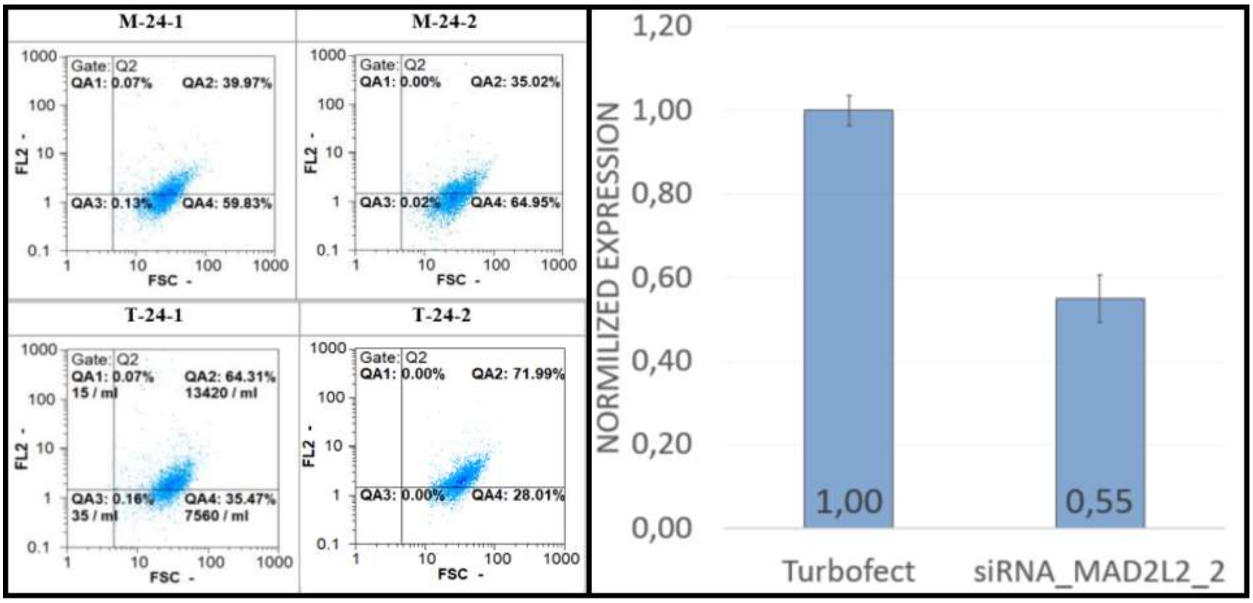

Figure S3. MAD2L2 protein knockdown with siRNA_MAD2L2. MAD2L2 was stained with primary (Recombinant AntiMad2L2/REV7 antibody [EPR13657], Abcam, UK) and secondary antibodies (Alexa Fluor 594 goat anti-rabbit IgG (H+L), Invitrogen, USA). A - Dot plot generated by the Flomax software; forward side scatter is along the X axis, fluorescent intensity, FL2, is along the y axis. M-24-1, M-24-2 – cells with knockdown of *MAD2L2,* 24 h after transfection, T-24-1, T-24-2 - control cells exposed to Turbofect only, 24 h after transfection. B – Proportion of MAD2L2-positive cells after *MAD2L2* knockdown. Mean and 95% CI. * $p < 0.05$.

Verification of Cas9-SCAI expression

Cas9-SCAI expression was confirmed by staining SCAI with primary (recombinant Anti-SCAI antibody [EPR4128] (ab124688), Abcam, UK) and secondary antibodies (Alexa Fluor 594 goat anti-rabbit IgG (H+L), Invitrogen, USA) 48 h after transfection with the Cas9-SCAI vector. Analysis of 62,959 cells transfected with Cas9-SCAI and 14,577 cells transfected with Cas9 only (control) confirmed that SCAI exhibits nuclear localization (S6 Fig) and its expression level is 12% higher in cells transfected with Cas9-SCAI (p=0.0001).

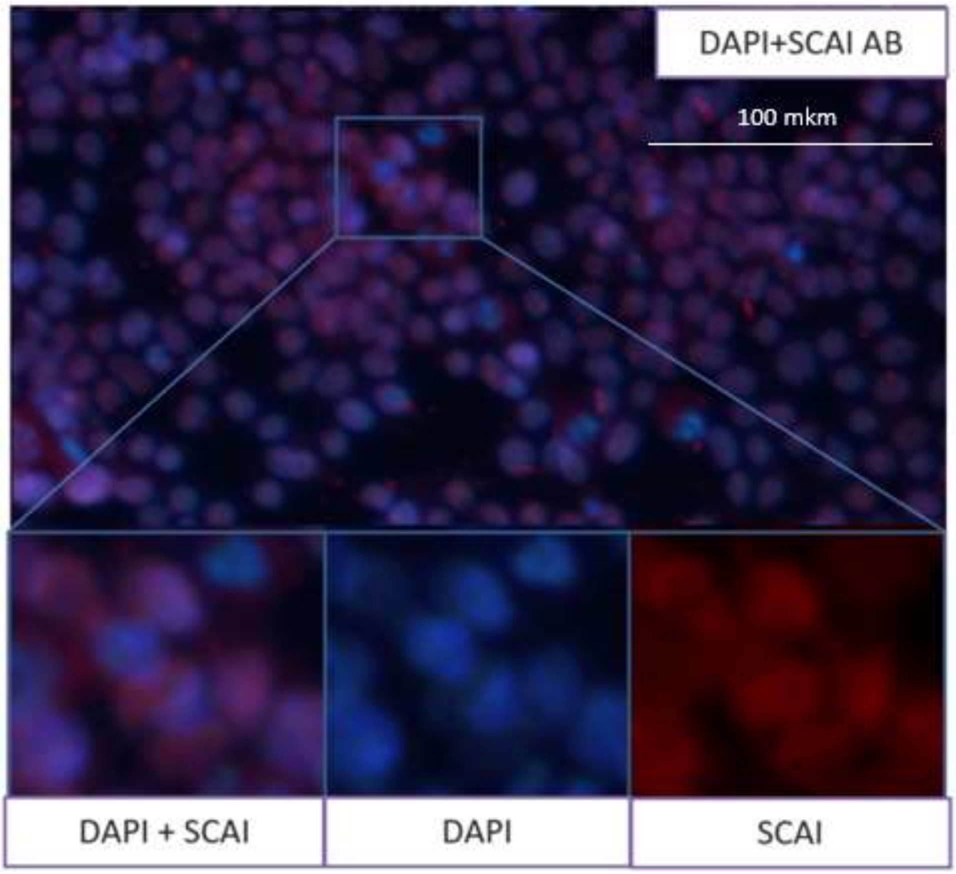

Figure S4. SCAI is localized in the nuclei of the transfected HEK293 cells stained with Anti-SCAI antibodies (SCAI AB) and DAPI.

SCR7: choosing optimal time and concentration

SCR7 which inhibits ligase IV was added to transfected cells at the time of transfection or 24 h before. Cells were transfected with the Cas9 to restore the *eGFP* gene by HDR. The number of GFP+ cells was 1.8 times higher (p=0.009) when exposed to SCR7 simultaneously with Cas9-sgRNA/ssODN transfection, while the administration of SCR7 24 h prior to transfection even reduced the number of successfully edited cells (by 2.4 times, p=0.009) (S4 Fig).

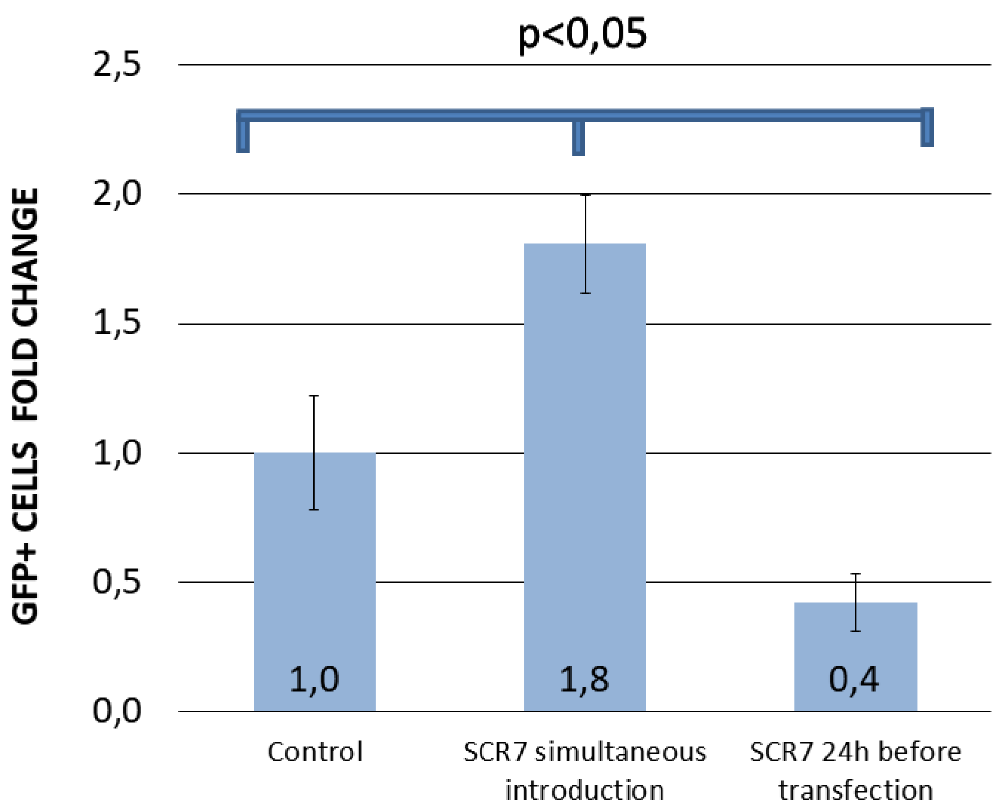

Figure S5. Influence of SCR7 added at different time points on the number of GFP+ cells after genome editing by Cas9-sgRNA/ssODN. Mean and 95% CI.

Next we added SCR7 at 4 different concentrations simultaneously with transfection. All of them were efficient in increasing the HDR efficiency (S5 Fig), there were no differences between different concentrations ($p > 0.4$). There was also no difference in cell survival (data not shown); the concentration of 1 µM was chosen as the most frequently used by other authors.

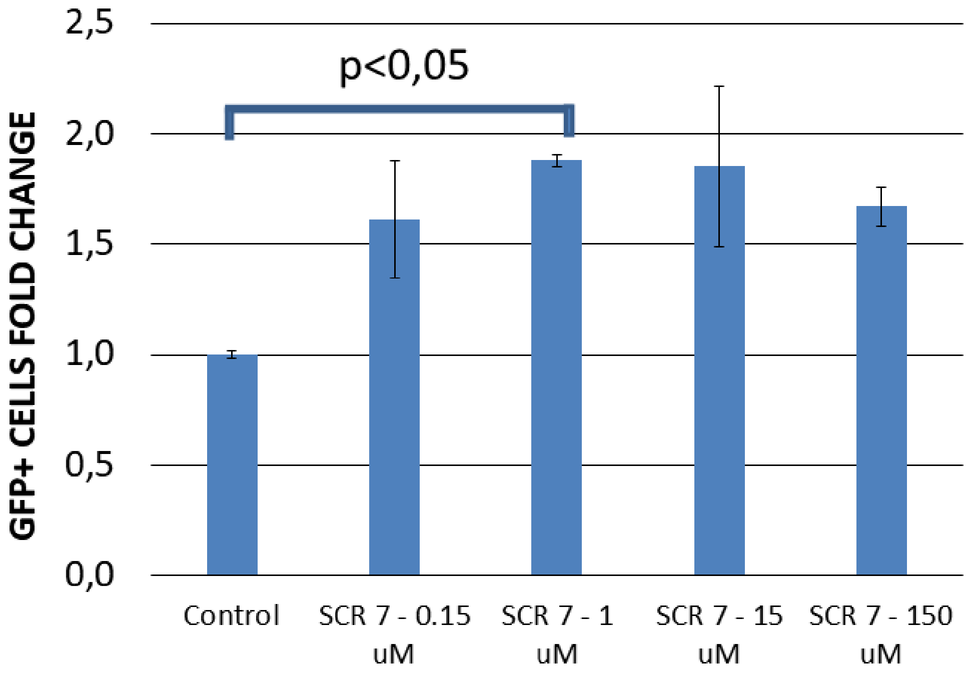

Figure S6. Influence of SCR7 added at different concentrations on the number of GFP+ cells after genome editing by Cas9-sgRNA/ssODN. Mean and 95% CI.

Supplementary Methods

Microscopy

Microscopy was performed using an Axio vert. A1 fluorescence microscope (Carl Zeiss, Germany) and Zen2.3 software.

SCAI expression was analyzed using a Leica TCS SP8 Confocal Laser Scanning Microscope (Leica Microsystems, Germany). Images were processed and analyzed using the open-source software CellProfiler version 3.0.0. Cell nuclei and cytoplasm were identified, after which the percentage of positive cells and fluorescence intensity in each cell were measured.

Immunostaining

Harvested cells were washed with the Dulbecco's Phosphate-Buffered Saline (DPBS), then transferred into 1.7 ml tubes and fixed with 4% formaldehyde, permeabilized with 0.25% Triton X100 in PBS and blocked with 1% BSA in PBST. After that, the cells were incubated for 30 min at room temperature with primary antibodies (Recombinant Anti-Mad2L2/REV7 antibody [EPR13657], Abcam, UK, 1:70; or Recombinant Anti-SCAI antibody [EPR4128] (ab124688), 1:160). Then the cells were washed with DPBS and incubated for 30 min with secondary antibodies (Alexa Fluor594 goat anti-rabbit IgG(H+L), Invitrogen, USA; 1:150). After washing with DBPS, the cells were analyzed by flow cytometry.

Table S1. Oligonucleotides used in the study

Types of oligos	Description	Sequence
**Primers**	for sequencing of *eGFP* locus	F 5’-ACGTAAACGGCCACAAGTTCA-3’<br>R 5'-CTGCCGTCCTCGATGTTGT-3'
	for cloning of *eGFPmut* cds in lentiviral vector	F 5’-GCCTGCTAGCGAGGGCAGAGGAAGTCTTCTAA-3’<br>R 5'-CTTTGTCGACTCACTTGTACAGCTCGTCCATGC CGAGAGTGA-3’
	for cloning of *TurboRFP* cds in lentiviral vector	F 5’-GTTTGGATCCGCCACCATGGTGGGTGAGGATAG CGT-3’<br>R 5'-GTTTGCTAGCGCCTCTGTGCCCCAGTTTGCTAG GGAGGT-3’
	for RT-PCR of *MAD2L2* gene	F 5’-CACTGCGTCAAGCCACTCCT-3’<br>R 5’-CAGCGAGTCTGAGCTGATGG-3’
	for reference house-keeping gene *TFRC*	F 5’-TCCTTGCATATTCTGGAATCCC-3’<br>R 5’-ATCACGAACTGACCAGCG-3’
	for reference house-keeping gene *TBP*	F 5’-CGGAGAGTTCTGGGATTGTAC-3’<br>R 5’-GTGGTTCGTGGCTCTCTTATC-3’
	for reference house-keeping gene *B2M*	F 5-’CTGCCGTGTGAACCATGTGA-3’<br>R 5’-CAATCCAAATGCGGCATCTTC-3’
	For RT-PCR of *SCAI* gene	F 5’-CAGAGGTTCTGGTGATAGCAGTC-3’<br>R 5’-CTGCCACTGCTTCTGTCCAT-3’
**Linker sequence**	For fusion of *Cas9* and *SCAI*	NT: 5’-TCCGGCTCCGAGACCCCCGGCACCTCCGA GTCCGCCACCCCCGAGTCC-3’<br>AA: SGSETPGTSESATPES
**gRNA**	guide RNA complementary a part of *eGFP* coding sequence with mutation p.337delG	5’-TGTCGCCCTCGAACTTCACT-3’<br>3-’ACAGCGGGAGCTTGAAGTGA-5’
**ssODN**	single-stranded oligodeoxynucleotide for	5’-TTCTTTAAGGACGACGGCAACTACAAGACCCGCGCCGAGGTGAAGTTCGAGGGCGACACCCTGGTGAACCGCATCGAGCTGAAGGGCATCGACTTCAAGGAGGACGGCAACATCCTCGGG-3’

	homology directed repair	
**siRNAs**	siRNA_MAD2L 2_1	5’-CCUCGACUUCUCAGCCUCCUU-3’<br>3’-UUGGAGCUGAAGAGUCGGAGG-5’
	siRNA_MAD2L 2_2	5’GUGCUCUUAUCGCCUCUGUdTdT3’<br>3’dTdTCACGAGAAUAGCGGAGACA5’
	siRNA_MAD2L 2_3	5’-GGAUGACCACGCUCACACGdTdT-3’<br>3’-dTdTCCUACUGGUGCGAGUGUGC-5
	siRNA_flu	5’FAM –AGGUCGAACUACGGGUCAAdTdC-3’<br>3’-dGdAUCCAGCUUGAUGCCCAGUU-FAM5’
